# The molecular and biochemical insight view of grape seed proanthocyanidins in ameliorating cadmium-induced testes-toxicity in rat model: implication of PI3K/Akt/Nrf-2 signaling

**DOI:** 10.1042/BSR20180515

**Published:** 2019-01-15

**Authors:** Nazima Bashir, Kalist Shagirtha, Vaikundam Manoharan, Selvaraj Miltonprabu

**Affiliations:** 1 Department of Zoology, Annamalai University, Chidambaram 608002, India; 2 Department of Biochemistry, St. Joseph’s College, Cuddalore 607001, India; 3 Department of Zoology, Faculty of Science, University of Madras, Chennai 600025, India

**Keywords:** antioxidant, cadmium, grape seed proanthocyanidins, Nrf2/HO-1, oxidative stress, testes

## Abstract

The present study aims to evaluate the protective effect of grape seed proanthocyanidins (GSP) on cadmium (Cd)-induced testicular apoptosis, inflammation, and oxidative stress in rats. A total of 24 male Wistar rats were divided into four groups, namely control, GSP (100 mg/kg BW), Cd (5 mg/kg BW), and Cd+GSP. Cd-treated rat testes exhibited a significant increment in oxidative stress mediated inflammation and apoptosis. Pre-administration of GSP exhibit significant protection against the apoptotic and inflammatory damages elicited by Cd and uphold the intercellular antioxidant status in testes. Histological changes were studied and the immunohistochemical staining for caspase 3, HSP70, and eNOS protein expressions were also analyzed to justify the protective action of GSP. Furthermore, GSP prevented DNA damage, and enhanced the expression of antioxidant responsive elements Nrf2/HO-1 by PI3K/Akt-dependent pathway. Therefore, our results suggest that GSP acts as a multipotent antioxidant entity against Cd-induced oxidative testicular toxicity in rats.

## Graphical abstract

**Graphical Abstract:**
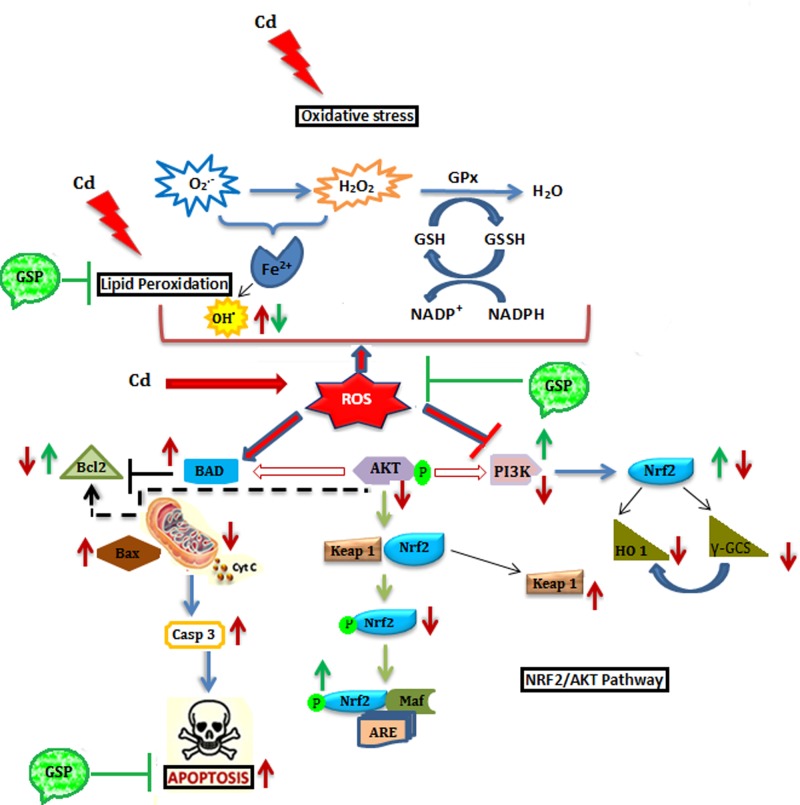
Mechanism of GSP protection against Cd testes toxicity

## Introduction

Cadmium (Cd) is considered as a potent reproductive toxicant, and has severe toxic effects in testis of rats and humans associated with the male infertility and deprived semen quality [1]. Cd brings testicular changes at various stages of growth and maturity which comprised severe edema, hemorrhage, necrosis and atrophy, as well as reduction in counts and motility of sperm and decreased concentrations of testosterone in plasma and testes [2]. Cd has been found to induce the non-reversible tissue necrosis at relatively low concentrations [3] and alters gene expression in testis even at non-toxic doses of Cd. Cd has potent estrogen and androgen like activities *in vivo* and *in vitro*, by directly binding to estrogen and androgen receptors [4].

The pathogenesis of testicular damage following Cd exposure are generally attributed to oxidative damage which cause damage to cells, stimulates free radical production, resulting in oxidative deterioration of lipids, proteins, and DNA. Cd exposure is one of the main causes of human prostate and testicular cancers due to the elevated levels of Cd in prostatic tissues and Leydig cell adenomas in the testis and epithelial cell adenomas in the ventral prostate of rats [5]. High levels of Cd in the modulation of male reproductive system are in seminal fluid which is associated with asthenozoospermia in infertile males. Based on variability of testicular damage, several mechanisms of Cd-induced testicular toxicity have been proposed. The physical and chemical properties of the Cd^+2^ ions, specifically its similarities to calcium and zinc put forth the oxidative stress of Cd. Cd substitutes the calcium or zinc in crucial physiological processes that are mediated by these ions, resulting in the activation and/or inhibition of several signaling pathways [6]. Another possible mechanism of Cd-mediated toxicity has been associated with the decreased Leydig cell viability leading to the decreased testosterone secretions, which stimulates the release of gonadotrophin releasing hormone from the hypothalamus [7].

Cd-induced toxicity to the testis involves the disruption of the blood–testes barrier (BTB) via specific signal barrier with specific signal transduction pathways and signaling molecules like p38 mitogen-activated protein kinase and Cd-associated capacity to induce the oxidative stress and apoptosis [8]. Oxidative stress is a major menace in Cd toxicity and is mediated by excess production of reactive oxygen species (ROS) which prompted our interest toward the antioxidants as possible agents for the management of Cd-induced reproductive toxicity. In this context, it was hypothesized that grape seed proanthocyanidins (GSP) might be useful since proanthocyanidins present in grape seeds serves as an effective free radical scavenger(s) and antioxidant(s).

The GSP are phenolic compounds with a structural diversity and complexity as compared with those in other plants or plant-derived foods such as apple, pear, and cocoa. GSP consist of −15% (+) catechin, (−) epicatechin; 80%(−) –epicatechin of 3-*O*-gallate, dimers, trimers, tetramers, and their gallates and 5% of pentamers, hexamers, heptamers, and their gallates [9]. GSP is one of the most powerful antioxidant and it protects cells from damage by controlling the oxidative damage by reducing the tissue injury and maintains antioxidant status and reduces the release of pro-inflammatory mediators [10]. GSP has several pharmacological effects which include antioxidative, anticarcinogenic, cardioprotective, and hepatoprotective. GSP exhibits more antioxidant properties than vitamin C, vitamin E, and β-carotene, which can also regulate cell signaling pathways, associated with apoptosis and inflammation [11]. We conjectured that GSP could attenuate Cd induced reproductive damage at molecular level, as no scrupulous *in vivo* studies have been performed previously. The reported properties of GSP warrants investigating the reproductive modulatory potential of GSP in a rat model of Cd-induced oxidative testicular dysfunction and to understand the complex mechanism behind the reprotoxic complications for developing efficient therapeutic strategy.

## Materials and methods

### Chemicals

GSP containing approximately 54% dimeric, 13% trimeric procyanidins, and 7% tetrameric proanthocyanidins were obtained from Pharmacol Gignos, Bangalore, India. Cd was given as Cadmium chloride (CdCl_2_) and all other chemicals were obtained from Sisco Research Laboratory, Andheri, Mumbai, India. BSA was purchased from Sigma Chemical Co., St. Louis, MO, U.S.A. Nrf2, HO-1, γGCS, NQO1, Keap-1, Bcl-2, Bad, caspase-3, and cytochrome *c*, anti-PI3k, p-PI3k anti-Akt, p-Akt antibodies were purchased from Santa Cruz Biotechnology, Inc, U.S.A., and goat anti-rabbit, anti-mouse, and rabbit anti-goat secondary antibodies were purchased from Genei, Bangalore, India. All other chemicals and solvents were of certified analytical grade and purchased from S.D. Fine Chemicals, Mumbai or Himedia Laboratories Pvt. Ltd., Mumbai, India. Reagent kits were obtained from Span Diagnostics, Mumbai, India.

### Animals

The present study used 24 healthy adult male albino Wistar rats weighing 210 ± 10 g. The animals were purchased from the Central Animal House, Faculty of Medicine, Annamalai University, Annamalai Nagar. They were kept in well-ventilated plastic cages under standard laboratory conditions. The animals were housed in a laboratory-controlled environment (25°C, 50% humidity) and the lighting schedule was maintained at 12 h of light per day. Feed (Lipton India Ltd, Mumbai, India) and water were provided *ad libitum.* All animal experiments were conducted in accordance with the Ethical Norms on Animal Care and use approved by the Institutional Animal Ethical Committee (IAEC) of Rajah Muthiah Medical College and Hospital (Reg. No. 1020/2013/CPCSEA), Annamalai University, Annamalai Nagar, India.

### Experimental design

In this experiment, a total of 24 rats were used. Six rats were used in each group: group I, normal control rats treated with vehicles alone daily for 4 weeks. Group II, normal rats treated with GSP dissolved in saline with a daily dose of 100 mg/kg for 4 weeks [12]. Group III, rats were orally treated with Cd in saline with a daily dose of 5 mg/kg body weight for 4 weeks which was 1/15 of the oral LD_50_ values in rats [12]. Group IV rats orally pre-administered with GSP (100 mg/kg) at an interval of 90 min and then administered with Cd in normal saline with a daily dose of 5 mg/kg body weight for 4 weeks.

At the end of the experimental period, rats were fasted overnight and all the rats were anesthetized under ketamine (75 mg/kg, i.p) using sterile conditions and then killed by cervical decapitation. Blood was collected; serum and plasma were separated by centrifugation. The final body weight, organ weight of the rats was recorded. The testes were excised, removed the connective tissues, weighed and homogenized in 100 mM potassium phosphate buffer containing 1 mM EDTA, pH 7.4 using a Potter–Elvehjem type homogenizer and centrifuged at 12000×***g*** for 30 min at 4°C. The supernatant was collected and used for various biochemical assays. Testes were collected at 24 h after Cd intoxication. Testes were divided in two parts: left one was kept at −80°C for Western blotting and apoptosis study. The right one was used for the histological and immunohistochemical studies. The testes were fixed in Bouin’s solution for 1–2 days, embedded in paraffin by routine method, serially sectioned at 5-µm thickness, and stained in Hematoxylin/Eosin for light microscopic evaluation.

### Light microscopical study

For qualitative analysis of testicular histology, the testes samples were fixed for 48 h at 10% formal-saline and dehydrated by passing successfully in different mixtures of ethyl alcohol and water, cleaned with xylene, and embedded in paraffin. Sections of tissue (5–6 μm thick) were prepared by using a rotary microtome and stained with Hematoxylin and Eosin and in neutral deparaffinated xylene (DPX) medium for microscopic observations.

### TEM

For TEM, the rat testes were fixed with 5% glutaraldehyde in 0.1 M phosphate buffer for 24 h. Samples from four rats/dose group were dehydrated in a series of acetone and embedded in Epon 812 by standard procedures. Eighty nanometer ultrathin sections were prepared and stained with both uranyl and lead citrate. The results were assessed using a JEM 1200 electron microscope (JEOL Ltd, Japan).

### Immunohistochemical analysis of HSP70, eNOS, and caspase 3

The paraffin-embedded testis were cut into 5-µm sections and mounted on positively charged slides for caspase-3, eNOS, iNOS, and HSP-70. Sections were dewaxed, rehydrated, and autoclaved at 120°C for 10 min in 10 mM citrate buffer (pH 6). After washing with PBS, endogenous peroxidase was blocked using 0.3% H_2_O_2_ in methanol for 15 min. Slides were washed in PBS again and blocking was performed by adding blocking buffer and incubated for 30 min at room temperature. Primary monoclonal and polyclonal antibodies for caspase-3 (Cat. No. PAI-29157, Thermo Fisher Scientific Co., U.S.A.), eNOS, iNOS (Santa Cruz Biotechnology Sc. 654), and HSP-70 (Stressegen, Canada, 1:200) were added after diluting with PBS (2 g/ml and 1:1000, respectively) and incubated for 30 min. The slides were washed three times for 3 min each with PBS. Biotinylated polyvalent secondary antibody (Cat No. 32230, Thermo Scientific Co., U.K.) was applied to tissue sections and co-incubated for 30 min. The slides were washed three times for 3 min each with wash buffer. The reaction was visualized by adding metal enhanced DAB Substrate Working Solution to the tissue and incubated for 10 min. The slides were washed two times for 3 min each with wash buffer. Counterstaining was performed by adding adequate amount of Hematoxylin stain to the slide to cover the entire tissue surface. Immunohistochemical staining was scored in a semiquantitative manner to determine the differences between the control group and the experimental groups. The intensity of the staining was recorded as weak (+/−), mild (+), moderate (++), and strong (+++). This analysis was performed in at least ten tubuli per testicular section, in five sections from each animal at 400×.

### Western blotting analysis

Western blotting was performed using testicular lysates. In brief, protein extracts from each sample were added to a gel loading buffer (100 mM Tris, pH 6.8, 20% glycerol, 200 mM DTT, 4% SDS, 0.03% Bromophenol Blue) boiled for 5 min. Proteins (50 µg/sample) in loading buffer were subjected to electrophoresis in 10–15% SDS/polyacrylamide gel for 3 h. The gel was transferred electrophoretically on to a PVDF membrane (Immobilon-P; Millipore Corp., Bedford, Massachusetts, U.S.A.) blocked with 5% non-fat powdered milk in Dulbecco’s PBS (DPBS) overnight at 4°C. The membranes were incubated for 2 h with the following antibodies: Nrf2, HO-1, NQO1, γGCS, Keap-1, anti-PI3k, p-PI3k, anti-Akt, and p-Akt Bcl-2, Bad, Caspase-3, Bax, and cytochrome *c*. β-Actin and Lamin B1 was used as loading control. After washing in DPBS containing 0.05% Tween-20 four times for 10 min each, the membranes were incubated with goat anti-rabbit or goat anti-mouse IgG antibody for 2 h. The membranes were then washed for four times in DPBS containing 0.05% Tween-20 for 10 min each, followed by signal development using an ECL detection kit from Pierce (Pierce Biotechnology, Rockford, IL).

### Determination of ROS in testes

The amount of ROS in testes was measured using 2′,7′-dichlorofluorescin diacetate (DCF-DA), which is converted into highly fluorescent DCF by cellular peroxides (including hydrogen peroxide). The assay was performed as described by us previously. Briefly, the testes were homogenized in 1 ml of ice-cold 40 mM Tris/HCl buffer (pH 7.4) and dilute (0.25%) with the same buffer and placed on ice. The samples were divided into two equal fractions. In one fraction, 40 µl of 1.25 mM DCF-DA in methanol was added for ROS estimation. Another fraction, to which 40 µl of methanol was added, served as control autofluorescence. All the samples were incubated for 15 min in 37°C water bath. Fluorescence was determined at 488 nm excitation and 525 nm emission using fluorescence plate reader.

### Determination of lipid peroxidation and oxidative stress markers

Lipid peroxidation was estimated spectrophotometrically by measuring thiobarbituric acid reactive substances and lipid hydroperoxides by the method of Niehiaus and Samuelsson [13], and Jiang et al. [14], respectively. Protein carbonyl content was determined by the method of Levine et al. [15]. The levels of conjugated diens were assessed by the method of Rao and Recknagel [16].

### Determination of non-enzymatic antioxidant levels in testes

Reduced glutathione (GSH) and total sulphydryl groups (TSH) were determined by the method of Ellman [17]. Vitamin C concentration was measured as previously reported Omaye et al. [18]. Vitamin E was estimated by the method of Desai [19].

### Determination of enzymatic antioxidant levels in testes

Superoxide dismutase (SOD) activity was determined by the method of Kakkar et al. [20]. The activity of catalase (CAT) was determined by the method of Sinha [21]. Glutathione peroxidase (GPx) activity was estimated by the method of Rotruck et al. [22] Glutathione S-transferase (GST) activity was determined by the method of Habig et al. [23]. Glutathione reductase (GR) was assayed by the method of Horn and Burns [24]. The estimation of glucose-6-phosphate dehydrogenase (G6PD) was carried out by the method of Beutler (1983) [25]. The total protein content of tissue homogenate was estimated as described previously by Lowry et al. [26].

### Assessment of membrane-bound ATPases

The sediment after centrifugation was resuspended in ice-cold Tris/HCl buffer (0.1 M) pH 7.4. This was used for the estimations of membrane-bound enzymes and protein content. The membrane bound enzymes such as Na^+^/K^+^-ATPase, Mg^2+^-ATPase, and Ca^2+^-ATPase activity was assayed by estimating the amount of phosphorous liberated from the incubation mixture containing tissue homogenate, ATP, and the respective chloride salt of the electrolytes [27–29]. Total protein content was estimated by the method described by Lowry et al. [26].

### Determination of Cd concentration

For determination of Cd concentration in testis, 1 g of tissue was digested with nitric acid in a microwave oven. After digestion, Cd was continuously preconcentrated and determined by flame atomic absorption spectrophotometry. A PerkinElmer 5000 atomic absorption spectrometer furnished with a Cd hollow-cathode lamp (lamp current :4 mA) was used to determine the Cd concentration. The instrument was set at 228.8 nm with a slit width of 0.5 nm. The acetylene flow rate was 2.0 l/min and an air flow rate of 17.0 l/min was employed to ensure an oxidizing flame.

### Determination of epididymal sperm count and motility

Epididymis sperm count and sperm progressive motility were evaluated by the method of Linder et al. [30]. Accordingly, epididymal spermatozoa were obtained by mincing the epididymis with anatomical scissors in 5 ml of physiological saline and incubated at 32°C for 2 min. An aliquot of this solution was placed in Neubauer hemocytometer and motile sperm were counted by using a microscope at 400× magnification. Non-motile sperm numbers were first determined, followed by counting of total sperm.

Sperm motility was expressed as a percent of motile sperm of the total sperm counted. Percentage of morphologically abnormal spermatozoa was determined by the method described by Evans and Maxwell [31]. According to this method, slides were prepared with Wells and Awa stains for morphological examination and 1% Eosin B and 5% Nigrosine in 3% sodium citrate dehydrate solution for live/dead ratio. A total of 400 sperm cells were counted on each slide under a light microscope at 100× magnification.

### Enzyme analysis

The aspartate aminotransferase (AST), alanine aminotransferase (ALT), acid phosphatase (ACP), alkaline phosphatase (ALP), and lactate dehydrogenase (LDH) by using UV kinetics methodology of the commercial diagnostic kit (Stanbio Co., Spain).

### Hormone assays

The hormones follicle stimulating hormone (FSH), leutinizing hormone (LH), prolactin, and testosterone were measured using the Vidas parametric system. The procedure was as described in the manufacturers’ manual (Diomeriux, MO, U.S.A.). All samples for a given experiment were performed within the same assay. Intra- and inter-assay coefficients of variation for the rat assay were 5 and 11%, respectively. Δ^5^ 3β-hydroxysteroid dehydrogenase (3β-HSD) and 17β-hydroxysteroid dehydrogenase (17β-HSD) activities were measured spectrophotometrically.

### Cytokines quantitation

The serum cytokines quantitation was assessed by ELISA, using commercial kits for interleukins (IL-1, IL-6, IL-10), tumor necrosis factor-α (TNF-α), interferon-γ (IFN-γ), and nitric oxide (NO) (eBioscience, San Diego, CA, U.S.A.), according to the manufacturer’s instructions. The results were expressed as µg/ml serum to IFN-γ and pg/ml serum to other parameters.

### The comet assay

Testes were dissected from rats, and a cell suspension containing primarily Sertoli cells and spermatogonia was isolated with multiple digestion steps. The comet assay was slightly modified from the original protocol described by Singh et al. [32]. Briefly, the fully frosted slides were pre-coated on each end with 100 µl of 0.8% agarose in PBS (pH 7.4), covered with a 22-mm × 22-mm glass coverslip and left at room temperature for 20 min. Then, 30 µl of the cell suspension was mixed with 70 µl of 1% low melting point agarose in PBS and maintained at 42°C on a dry-bath incubator. The mixture was immediately spread on to each end of a pre-coated slide and covered with a fresh glass coverslip. The process included lysing, denaturing, unwinding, electrophoresis, neutralizing, and staining. Finally, with a fluorescence microscope, 100 cells were counted at least twice for each slide. The comets were captured with an Olympus fluorescent microscope that was equipped with a CCD camera, and the images were quantitatively evaluated for the percentage of tail DNA, tail length (µm), tail migration, and olive tail migration using CASP software (Comet Assay Software Project 1.2.2) [33].

### Statistical analysis

Results were expressed as mean ± S.D. for a number of experiments (*n*=6). The statistical significance was evaluated by one-way ANOVA using SPSS version 16.0 (SPSS, Cary, NC, U.S.A.) and the individual comparisons were obtained by Duncan’s multiple range test (DMRT). A value of *P*<0.05 was considered to indicate a significant difference amongst groups.

## Results

### GSP effectively scavenges ROS in the testes of Cd-treated rats

**Figure 1:**
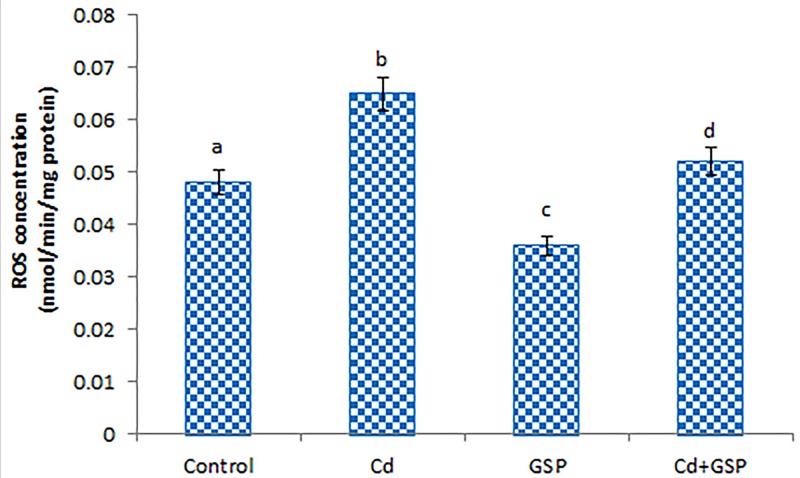
Effect of GSP on Cd induced ROS concentration in experimental rats Values are mean ± S.D. for six rats in each group. Values not sharing common superscript letters (a–d) differ significantly at *P*<0.05 (DMRT).

The concentration of ROS in the testes of experimental rats was shown in Figure 1. A significant (*P<*0.05) increase in the level of ROS was observed in the testes of Cd intoxicated rats when compared with the control rats. Pre-administration of GSP to Cd-treated rats shows a significantly (*P*<0.05) decreased level of ROS when compared with Cd alone treated rats. GSP alone treated rat also exhibited a significant (*P*<0.05) decrease in the level of testicular ROS when compared with the control rats.

### Effect of GSP on testes weight and testicular Cd concentration

The data in Table 1 show the effect of Cd and GSP on body weight, testicular weight, epididymal weight, caudal epididymal weight, and testicular Cd concentration in control and experimental rats. Cd treatment caused a significant (*P*<0.05) decrease in the body weight, epididymal and caudal epididymal weight with a significant (*P*<0.05) increase in testicular and Cd concentration in testis of rats when compared with the control rats. However, pre-administration of GSP to Cd intoxicated rats exerted a significant (*P*<0.05) restoration in Cd-induced alterations in testes, epididymal and caudal epididymal weight and testicular Cd concentration when compared with the Cd alone treated rats. No significant changes were observed in the GSP alone treated group of rats when compared with that of the control group.

**Table 1:** Effects of GSP on absolute reproductive organ weights in Cd-treated male rats

Groups	Control	Cd	GSP	Cd + GSP
Body weight gain (g)	12.16 ± 2.1^1^	6.42 ± 2.7^2^	13.0 ± 1.9^1^	13.26 ± 3.4^1,3^
Testes (g)	0.68 ± 0.21^1^	0.35 ± 0.13^2^	0.69 ± 0.24^1^	0.62 ± 0.27^1,3^
Epididymis (g)	0.17 ± 0.05^1^	0.08 ± 0.04^2^	0.15 ± 0.08^1^	0.14 ± 0.07^1,3^
Seminal vesicles (g)	0.30 ± 0.11^1^	0.21 ± 0.05^2^	0.27 ±0.09^1^	0.27 ± 0.12^1,3^
Prostate glands (g)	0.14 ± 0.09^1^	0.09 ± 0.04^2^	0.13 ± 0.14^1^	0.15 ± 0.11^1,3^

Values are given as mean ± S.D. from six rats in each group. Values with different superscript numbers (1–3) in the same column differ significantly at *P*<0.05 (DMRT).

### Effect of GSP on Cd induced alterations in testes marker levels

The data in Table 1 show the effect of Cd and GSP on testes ASL, ALT, ALP, ACP, and LDH levels in control and experimental rats. Cd treatment caused a significant (*P*<0.05) increase in testicular marker enzymes when compared with the control rats. However, pre-administration of GSP to Cd intoxicated rats exerted a significant (*P*<0.05) restoration in Cd-induced alterations in these marker enzymes when compared with the Cd-treated rats. No significant changes were observed in the GSP alone treated group of rats when compared with that of the control group.

### Effect of GSP on Cd induced changes in hormone levels

**Figure 2:**
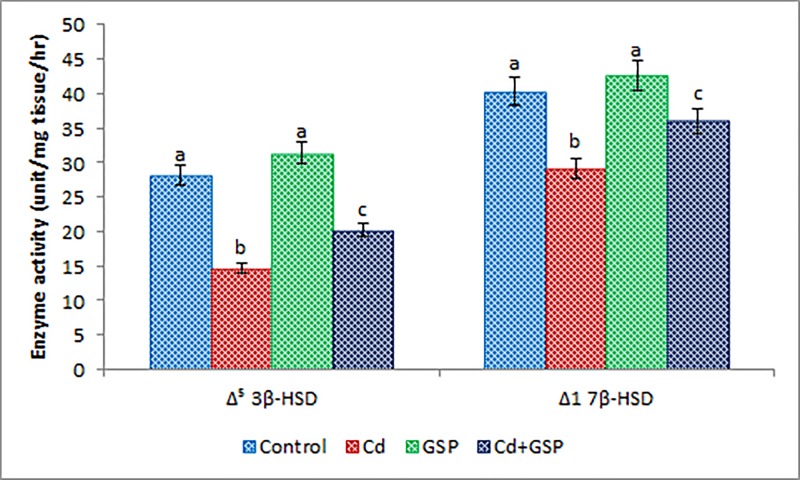
Effect of GSP on Cd-induced changes in steroidogenic enzyme activity in experimental rats Values are mean ± S.D. for six rats in each group. Values not sharing common superscript letters (a–c) differ significantly at *P*<0.05 (DMRT).

In Cd-treated rats, the levels of FSH, LH, and testosterone shown in Table 2, Δ^5^ 3β-HSD and Δ17β-HSD shown in Figure 2 were significantly (*P*<0.05) decreased with a significant (*P*<0.05) increase in the prolactin level (Table 2) when compared with the control rats. The decreased level of FSH, LH, and testosterone were significantly (*P*<0.05) increased in GSP pre-administered rats with significant decrease in prolactin level (Table 2) when compared with the Cd alone treated rats. GSP alone treated groups did not exemplify any changes in testicular steroidogenic enzymes and testosterone levels (Table 3).

**Table 2:** Effect of GSP on Cd induced changes in circulating hormones in experimental rats

Groups	Control	Cd	GSP	Cd+GSP
LH (IU/I)	9.08 ± 1.4^1^	3.3 ± 1.2^2^	8.9 ± 1.4^3^	6.9 ± 1.6^4^
FSH (IU/I)	11.2 ± 1.2^1^	4.9 ± 1.3^2^	10.3 ± 1.1^3^	7.2 ± 1.4^4^
Testosterone (nmol/I)	17.6 ± 2.4^1^	9.2 ± 2.2^2^	18.9 ± 2.3^3^	14.6 ± 2.1^4^
Prolactin (IU/I)	87.4 ± 3.8^1^	110 ± 8.4^2^	85.1 ± 6.2^3^	98.9 ± 2.8^4^

Values are given as mean ± S.D. from six rats in each group. Values with different superscript numbers (1–4) in the same column differ significantly at *P*<0.05 (DMRT).

**Table 3:** Effect of GSP on Cd-induced changes in testis biomarkers in experimental rats

Groups	Control	Cd	GSP	Cd+GSP
AST (1 U/g)	67.3 ± 1.02^1^	126.9 ± 1.34^2^	69.7 ± 1.31^3^	84.2 ± 1.72^4^
ALT (1 U/g)	33.5 ± 0.23^1^	68.1 ± 0.86^2^	32.8 ± 0.12^3^	43.02 ± 0.11^4^
ALP (1 U/g)	47.4 ± 0.18^1^	89.05 ± 1.15^2^	49.3 ± 0.22^3^	58.8 ± 1.16^4^
ACP (1 U/g)	10.6 ± 0.04^1^	23.4 ± 0.31^2^	9.8 ± 0.06^3^	14.6 ± 0.08^4^
LDH (1 U/g)	97.09 ± 1.61^1^	142.1 ± 1.28^2^	101.5 ± 1.49^3^	109.4 ± 1.16^4^

Values are given as mean ± S.D. from six rats in each group. Values with different superscript numbers (1–4) in the same column differ significantly at *P*<0.05 (DMRT).

### GSP curtailed the sperm abnormalities in the Cd-intoxicated rat testes

**Figure 3:**
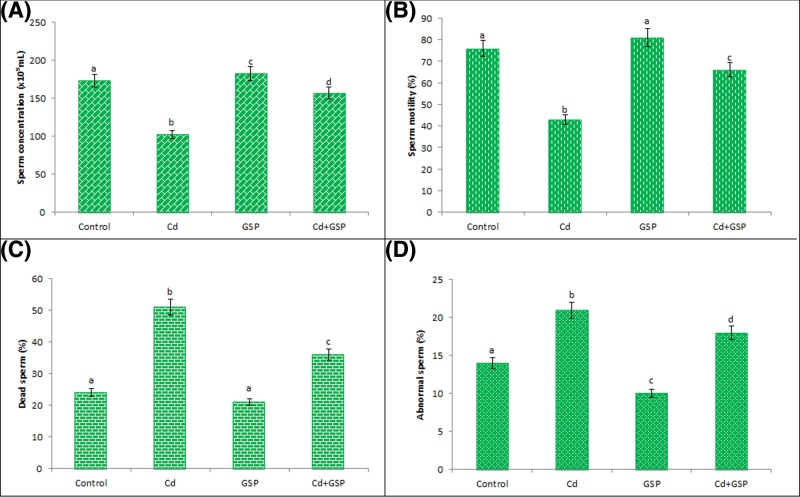
Effcet of GSP and Cd on Sperm parameters Effect of GSP and Cd on sperm parameters (**A**), sperm motility (**B**), dead sperm (**C**), and abnormal sperms (**D**) in experimental rats. Values are mean ± S.D. for six rats in each group. Values not sharing common superscript letters (a–d) differ significantly at *P*<0.05 (DMRT).

Epididymal sperm concentration, sperm motility, abnormal sperm rate, and live/dead count of sperms were shown in Figure 3. Cd intoxication showed a significant (*P*<0.05) decrease in epididymal sperm concentration (Figure 3A), sperm progress motility (Figure 3B), dead sperm count (Figure 3C), and increased abnormal sperm rate (Figure 3D). Pretreatment of GSP in Cd intoxicated rats brought a significant (*P*<0.05) improvement in epididymal sperm concentration, sperm motility, and live/dead count as well as a significant (*P*<0.05) reduction in the abnormal sperm rate. No significant change was observed in GSP alone treated rats in the level of live sperm count, epididymal sperm concentration, and sperm motility when compared with control rats.

### GSP suppresses the level of oxidative stress markers in the Cd-intoxicated testes

Table 4 shows the level of oxidative stress markers in control and experimental testes. Oxidative stress markers, such as TBARS, LOOH, PC, CD, and NO were significantly (*P*<0.05) increased in Cd intoxicated rats when compared with control rats. However, pre-treatment with GSP in Cd-treated rats significantly (*P*<0.05) decreased the level of oxidative stress markers when compared with Cd alone treated rats.

**Table 4:** Changes in the levels of oxidative stress markers in the testes of experimental rats

Groups	Control	Cd	GSP	Cd+GSP
TBARS (nmoles/g tissue)	22.92 ± 0.91^1^	34.39 ± 1.05^2^	23.34 ± 1.08^3^	27.01 ± 1.01^4^
LOOH (nmoles/g tissue)	0.91 ± 0.19^1^	2.01 ± 0.61^2^	0.94 ± 0.12^1^	1.07 ± 0.34^4^
PC (nmoles/mg protein)	1.81 ± 0.21^1^	3.51 ± 0.58^2^	1.72 ± 0.17^3^	2.11 ± 0.27^4^
CD (nmoles/mg protein)	1.95 ± 0.13^1^	4.70 ± 0.22^2^	1.85 ± 0.11^3^	2.52 ± 0.16^4^
NO (nmol per 100 mg tissue)	31.76 ± 3.43^1^	87.42 ± 6.76^2^	23.74 ± 2.34^3^	45.27 ± 2.71^4^

Values are given as mean ± S.D. from six rats in each group. Values with different superscript numbers (1–4) in the same column differ significantly at *P*<0.05 (DMRT).

### GSP augments the non-enzymatic antioxidant levels in the Cd-intoxicated testes

Table 5 depicts the effect of GSP on Cd induced changes of non-enzymatic antioxidant levels in control and experimental rats. There were significantly (*P*<0.05) decreased levels of GSH, TSH, Vitamin C and E in Cd intoxicated rats when compared with control group. Pre-administration of GSP significantly (*P*<0.05) increased the levels of GSH, TSH, Vitamin C and E near to normal control. No significant changes were observed in the non-enzymatic antioxidant levels of GSP alone treated group when compared with the control rats.

**Table 5:** Changes in non-enzymatic antioxidant status of testis tissue in experimental rats

Groups	Control	Cd	GSP	GSP + Cd
GSH (nmoles/mg protein)	4.46 ± 0.41^1^	2.27 ± 0.33^2^	4.51 ± 0.25^1^	4.18 ± 0.35^3^
TSH (nmoles/mg protein)	10.67 ± 0.68^1^	7.37 ± 0.37^2^	10.70 ± 0.58^1^	9.82 ± 0.36^3^
Vitamin C (µmol/mg tissue)	0.46 ± 0.18^1^	0.35 ± 0.19^2^	0.54 ± 0.12^3^	0.40 ± 0.11^4^
Vitamin E (µmol/mg tissue)	0.69 ± 0.15^1^	0.39 ± 0.23^2^	0.63 ± 0.17^1^	0.55 ± 0.20^3^

Values are mean ± S.D. for six rats in each group. Values not sharing common superscript numbers (1–4) differ significantly at *P*<0.05 (DMRT).

### GSP enhances the enzymatic antioxidant levels in the Cd intoxicated testes

Table 6 illustrates the activities of enzymatic antioxidants status in the testes of control and experimental rats. Cd-treated rats showed a significant (*P*<0.05) decrease in the activities of SOD, CAT, GPx, GST, GR, and G6PD when compared with control rats. Pre-treatment of GSP to Cd intoxicated rats significantly (*P*<0.05) increased the activities of SOD, CAT, GPx, GST, GR, and G6PD when compared with Cd-treated rats. GSP alone treated rats did not exhibit any changes in the antioxidant enzyme level when compared with control group.

**Table 6:** Effect of GSP on enzymatic antioxidant status of testis in experimental rats

Groups	Control	Cd	GSP	GSP+Cd
SOD	68.25 ± 2.15^1^	32.37 ± 1.08^2^	65.15 ± 1.12^3^	49.25 ± 2.15^4^
CAT	58.12 ± 2.03^1^	35.32 ± 2.29^2^	60.08 ± 1.41^3^	43.12 ± 1.34^4^
GPx	108.03 ± 3.81^1^	74.44 ± 3.15^2^	107.10 ± 2.32^3^	91.34 ± 2.05^4^
GR	97.79 ± 1.22^1^	75.16 ± 2.27^2^	99.13 ± 2.15^3^	86.24 ± 2.34^4^
GST	12.02 ± 0.13^1^	5.36 ± 0.11^2^	11.01 ± 0.23^3^	9.25 ± 0.11^4^
G6PD	9.09 ± 0.15^1^	3.19 ± 0.1^2^	9.01 ± 0.13^3^	6.25 ± 0.32^4^

Values are mean ± S.D. for six rats in each group. Values not sharing common superscript numbers (1–4) differ significantly at *P*<0.05 (DMRT). SOD – One unit of enzyme activity was taken as the enzyme reaction, which gave 50% inhibition of NBT reduction in 1 min/mg protein. CAT – µmol of H_2_O_2_ utilized/min/mg protein. GPx – µg of GSH consumed/min/mg protein. GST – µmol of CDNB–GSH conjugate formed/min/mg protein. GR – nmol of NADPH oxidized/min/mg protein. G6PD were expressed as nmol of NADPH formed/min/mg protein.

### The effect of Cd and GSP on membrane-bound ATPases

**Figure 4:**
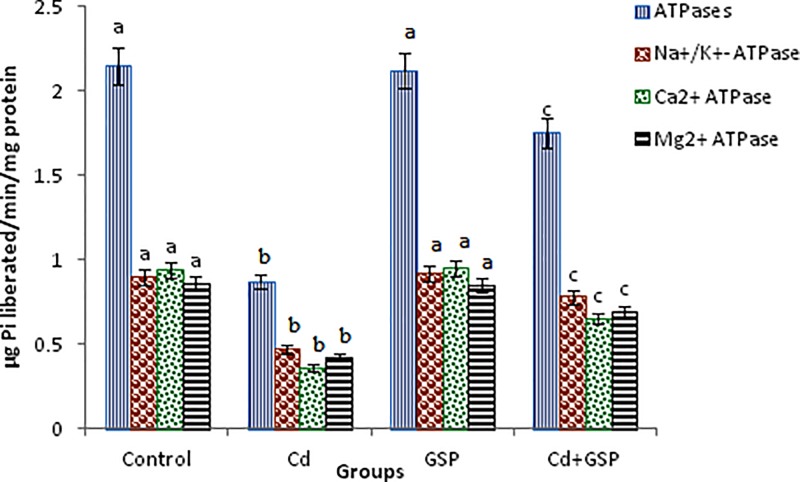
Effect of GSP on Cd-induced changes in ATPases of experimental rats in testis Values are mean ± S.D. for six rats in each group. Values not sharing common superscript numbers (a, b, c) differ significantly at *P*<0.05 (DMRT).

Figure 4 shows the activities of total ATPase, Na^+^/K^+^-ATPase, Mg^2+^-ATPase, and Ca^2+^-ATPase in the testes of control and experimental rats. The activities of these membrane-bound ATPases in the testes tissues of Cd-treated rats were significantly (*P*<0.05) decreased when compared with the control rats. Pre-treatment of GSP before Cd intoxication significantly (*P*<0.05) restored the levels of the entire membrane-bound ATPases when compared with Cd alone intoxicated rats. No significant changes observed between control and GSP alone treated rats.

### GSP effect on pro-inflammatory cytokines in Cd -ntoxicated testes

The effect of GSP on the serum level of pro-inflammatory cytokines in control and experimental rats are depicted in Table 7. The serum level of TNF-α, IL-1β, IL-6, IL-10, and IFN-γ was significantly (*P*<0.05) increased in Cd-treated rats when compared with control rats. On the other hand, pre-treatment with GSP showed a significant reduction in the Cd-induced increase in pro-inflammatory cytokines as compared with the Cd intoxicated rats. GSP alone treated group did not show any significant changes in these inflammatory markers when compared with control rats.

**Table 7:** Effect of GSP on IL-1, IL-6, TNF-α, IFN-γ, and IL-10 of testis in experimental rats

Groups	IL-1 (pg/ml)	IL-6 (pg/ml)	TNF-α (pg/ml)	IFN-γ (µg/ml)	IL-10 (pg/ml)
Control	24.41 ± 1.41^1^	32.10 ± 2.1^1^	52.01 ± 2.52^1^	61.25 ± 2.32^1^	58.15 ± 2.99^1^
Cd	65.33 ± 1.08^2^	60.66 ± 1.82^2^	86.26 ± 1.0^2^	95.32 ± 2.12^2^	79.32 ± 2.6^2^
GSP	26.11 ± 1.25^3^	30.09 ± 1.15^3^	53.02 ± 1.26^3^	63.15 ± 2.1^3^	56.21 ± 2.3^3^
Cd+GSP	39.9 ± 1.91^4^	48.03 ± 1.13^4^	66.3 ± 1.04^4^	74.01 ± 1.21^4^	65.33 ± 1.2^4^

Values are mean ± S.D. for six rats in each group. Values not sharing common superscript number (1–4) differ significantly at *P*<0.05 (DMRT).

### GSP stabilizes the protein expression of Bcl-2, Bad, Bax, Cyt-*c*, and Cas-3 in the Cd-intoxicated testes

**Figure 5:**
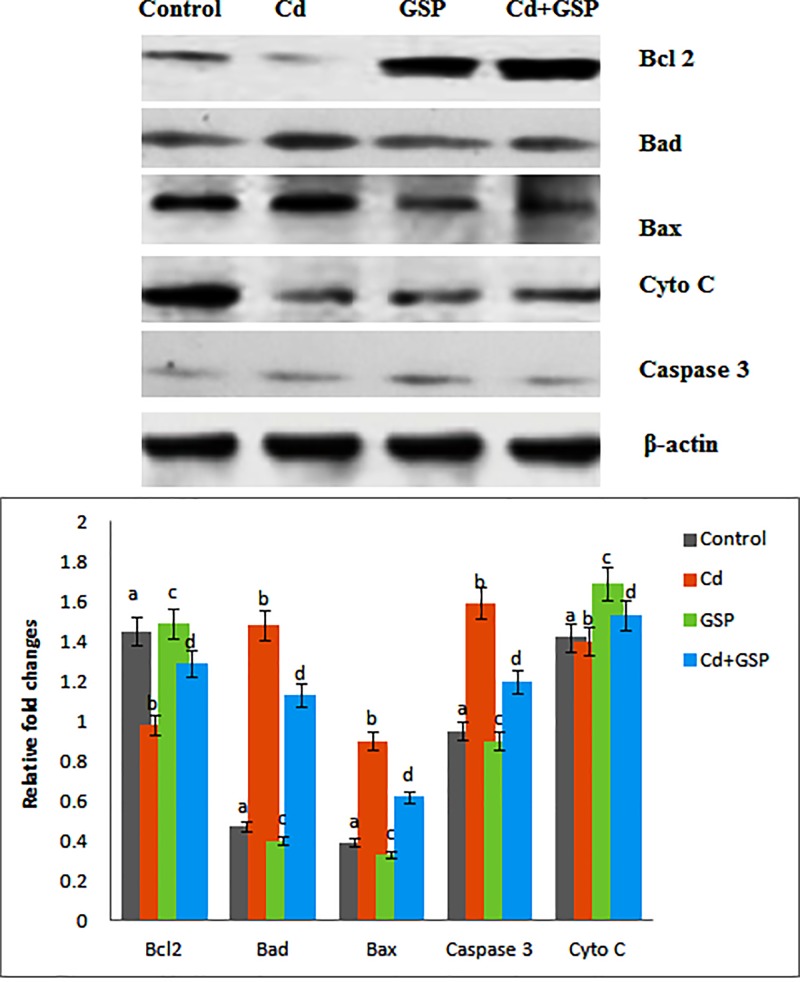
Effect of GSP and Cd on apoptotic markers Effect of GSP on Bcl-2, Bad, Bax, cytochrome *c*, and Caspase 3 protein expressions in the testes tissues of experimental rats by Western blot: effect of GSP on Lane 1. Control, Lane 2. Cd (5 mg/kg BW), Lane 3. GSP control (100 mg/kg bw) Lane 4. Cd (5 mg/kg BW) + GSP (100 mg/kg BW). Effect of GSP on Bcl-2, Bad, Bax, cytochrome *c*, and Caspase 3 protein band intensities scanned by densitometer. Values are mean ± S.D. for six rats in each group. Values not sharing common superscript letters (a–d) differ significantly at *P*<0.05 (DMRT).

The effect of pre-treatment of GSP on the levels of Bcl-2, Bad, Bax, Cyt-*c*, and Cas-3 in testes tissues of control and experimental groups of rats are demonstrated in Figure 5. The levels of Bcl-2, Bad, Bax, Cyt-*c*, and Cas-3 in control rats administered with GSP did not reveal any statistical difference when compared with that of control group of rats. Conversely, there was a significant (*P*<0.05) decrease in the level of Bcl-2 with a significant (*P*<0.05) increase in the levels of Bad, Bax, Cas-3, and Cyt-*c* in the testes of Cd-treated rats in comparison with control group of rats. Moreover, pre-treatment with GSP to Cd group of rats significantly (*P*<0.05) restored the altered levels to near normalcy when compared with Cd-treated group of rats.

### GSP effects on protein levels of Nrf2, Keap1, γ-GCS, NQO1, HO-1, anti-PI3k, p-PI3k, anti-Akt and p-Akt in Cd-intoxicated testes

**Figure 6:**
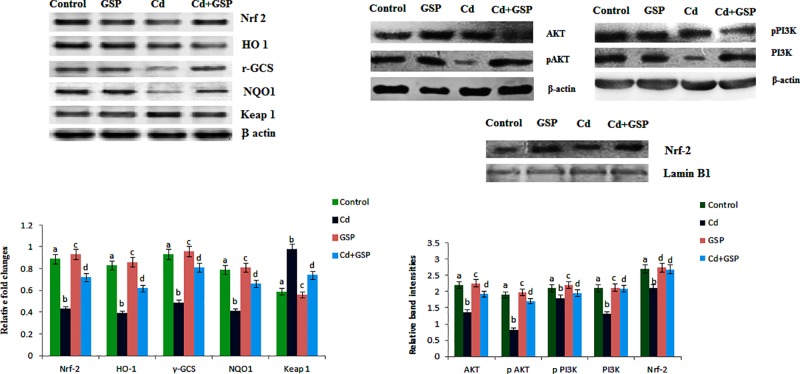
Effect of GSP and Cd on testes antioxidative markers Effect of GSP on Nrf2, Keap1, γ-GCS, NQO1, HO-1, anti-PI3k, p-PI3k, anti-Akt, and p-Akt protein expressions in the testes of experimental rats by Western blot: β-actin and Lamin B1 was used as an internal control. Lane 1. Control, Lane 2. Cd (5 mg/kg BW), Lane 3. GSP control (100 mg/kg bw) Lane 4. Cd (5 mg/kg BW) + GSP (100 mg/kg BW). Effect of GSP on Nrf2, Keap1, γ-GCS, NQO1, HO-1, anti-PI3k, p-PI3k, anti-Akt and p-Akt protein expressions in the testes scanned by densitometer: values are mean ± S.D. for six rats in each group. Values not sharing common superscript letters (a–d) differ significantly at *P*<0.05 (DMRT).

The effect of GSP on the protein levels in the testes tissues of control and experimental groups of rats are depicted in Figure 6. The protein levels of Nrf2, γ-GCS, NQO1, HO-1, anti-PI3k, p-PI3k, anti-Akt and p-Akt were significantly (*P*<0.05) decreased with a simultaneous elevation of Keap1 protein in the testes of Cd-treated group of rats. However, these altered protein levels were significantly (*P*<0.05) normalized in the Cd-treated group of rats with the pre-administration of GSP. On the other hand, GSP alone treated group also exhibit a significant difference on the protein levels of Nrf2, Keap1, γ-GCS, NQO1, HO-1, anti-PI3k, p-PI3k, anti-Akt and p-Akt in comparison with the control group of rats.

### Effect of GSP on DNA damage of testicular cells induced by Cd exposure

**Figure 7:**
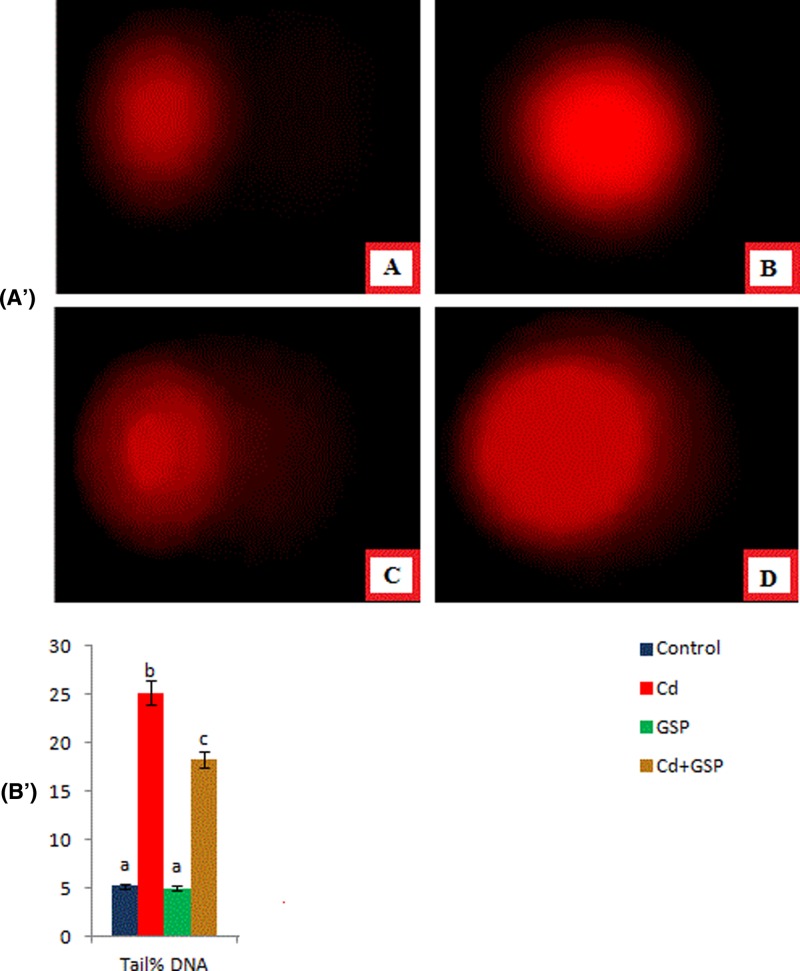
Effect of GSP on DNA damage of testicular cells induced by Cd intoxication (**A’**) The testicular DNA damage was measured by comet assay. (**A**) Control rats show no DNA migration. (**B**) GSP alone administered rats show no DNA migration. (**C**) Cd-treated rats show extensive DNA migration. (**D**) GSP pre-administered Cd intoxicated rats show minimal DNA migration. (**B’**) Graphical representation of Comet assay(% Tail DNA).values are mean ± S.D. for six rats in each group. Values not sharing common superscript letters (a–c) differ significantly at P<0.05 (DMRT).

Based on findings of the comet assay, considerable DNA damage was found in Cd-exposed rats. The % DNA in tail in the Cd-exposed group was higher than those of the control group. However, administration of GSP to Cd-exposed rats resulted in a decreased level of DNA damage (Figure 7) compared with the Cd-treated group. These results suggest that GSP can ameliorate DNA damage induced by Cd.

### Effect of GSP on testicular immunohistochemistry

**Figure 8:**
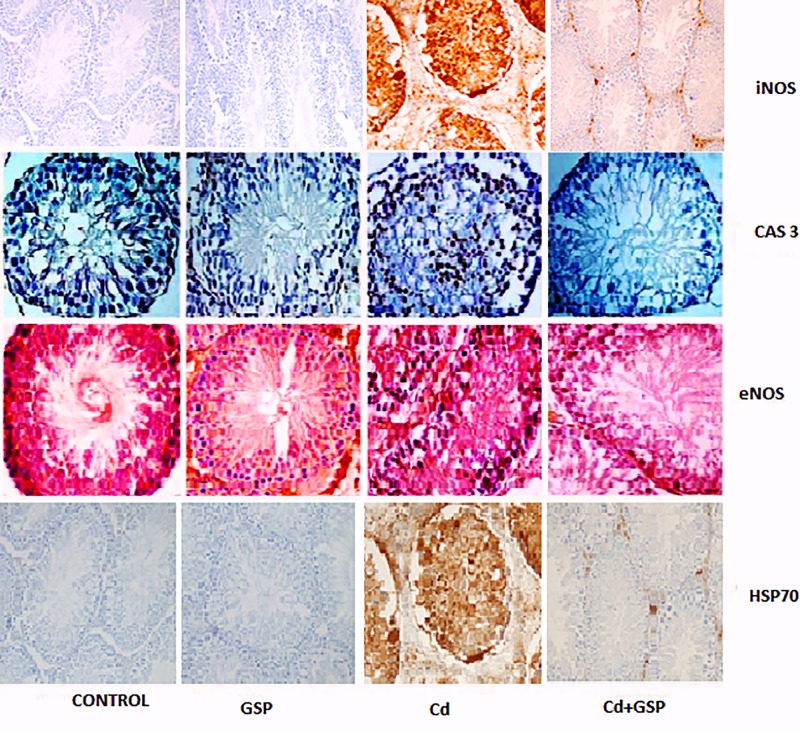
Effect of GSP and Cd on Immunohistochemical staining of iNOS, caspase-3, eNOS, HSP70 in rat testis Immunohistochemical staining of iNOS, caspase-3, eNOS, HSP70 in rat testis: Testis of control rats shows no immunoreaction. Testis of GSP-treated rats seems normal as control group. Testis of Cd-treated rats, the intensity of iNOS, caspase-3, eNOS, HSP70 immunostaining is predominant on spermatogonia and seminiferous tubules of treated group. Testis of Cd+GSP rats shows moderate immunoreaction.

As shown in Table 8 and Figure 8, testicular tissues from Cd-treated rats stained strongly resulted in significant increase in the immunoreactivity of iNOS, eNOS, HSP70, and caspase 3 in the cytoplasm of seminiferous tubular cells as compared with the control group. This was reversed in the GSP pre-treated Cd-treated group which showed significant reductions in the Cd-induced expression of the tested proteins in testicular tissue as compared with the Cd alone treated rats. There was no difference in the expression of eNOS, HSP70, and caspase 3 in the GSP group compared with the control group.

**Table 8:** Immunohistochemistry staining of control, GSP, Cd, Cd+GSP groups

Parameters	Control	GSP	Cd	Cd+GSP
Caspase 3	+	+	+++	++
eNOS	+	+	+++	++
iNOS	+	+	+++	+
HSP70	+	+	+++	+

Intensity of staining was recorded as weak (+/−), mild (+), moderate (++), and strong (+++).

### Histopathological examination of the testicular tissues

**Figure 9:**
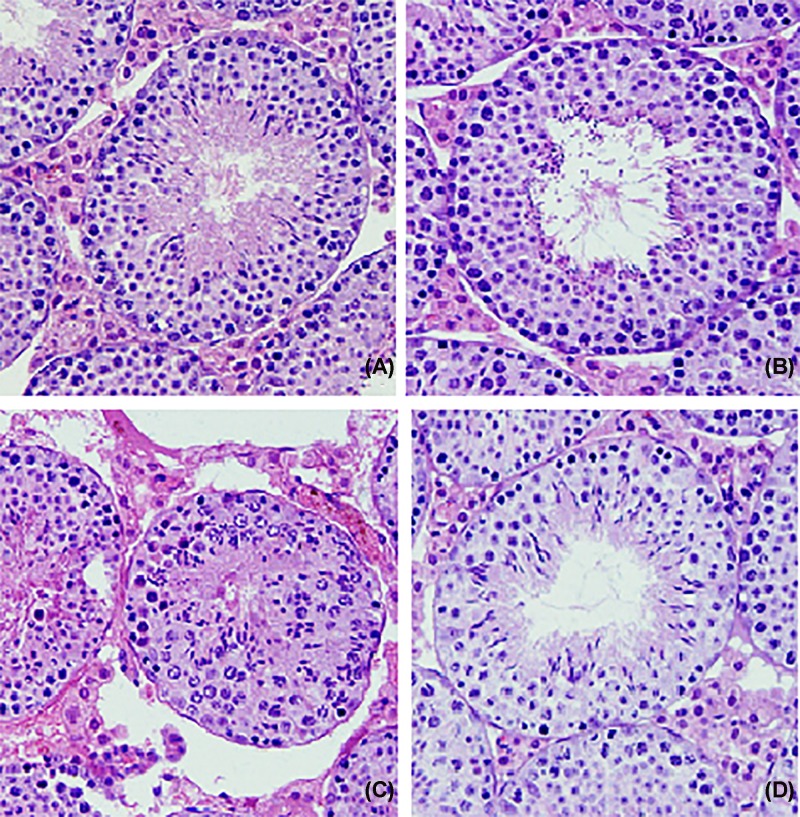
Effect of GSP and Cd on the histomrphology of rat testes Photomicrographs of rat testes from control and experimental rats (H & E, ×200): the testes from control and GSP (**A**,**B**) rats showed normal histological architecture of seminiferous tubules, Sertoli cell with spermatogenesis. (**C**) Section of Cd-treated rat displayed marked disorder of the normal architecture of the testicular tissues, where the seminiferous tubules swell and enlarge with focal hemorrhage and necrosis of Leydig cells. (**D**) Testis section of GSP pre-treated Cd-intoxicated rats show significant improvement of histological architecture with normal seminiferous, spermatocytes, and interstitial cells.

Histopathological observation of the testis is shown in Figure 9. The control (Figure 9A) and GSP group (Figure 9B) showed normal testicular structures, where each seminiferous tubule was lined with a germinal epithelium and spermatogenesis supported with scattered Sertoli cells. On the other hand, histopathological investigation of sections of the testes in Cd-treated rats (Figure 9C) showed disturbances in spermatogenesis process including degenerative changes in the testes with severe disintegration of spermatocytes, spermatogenic arrest with moderate tubular necrosis, and Leydig’s cell degeneration. Pre-treatment of GSP in Cd-treated rats (Figure 9D) revealed some improvements in spermatogenesis process as judged by the presence of all stages of sperm formation, quite normal testicular histoarchitecture and seminiferous tubules tightly connected together with inter-tubular connective tissue.

### Effect of GSP on electron microscopy of testicular tissue

**Figure 10:**
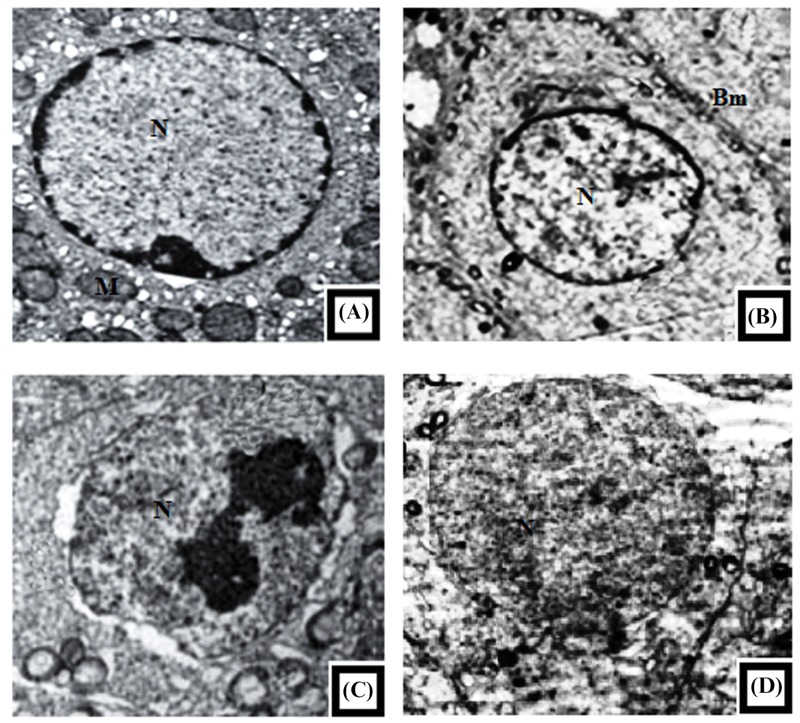
Representative TEM photographs from the testes of control and experimental rats (**A**) Normal histology of the control rat testis where seminiferous tubules, interstitial cells, and various stages of spermatogenesis are clearly visible (N-Spermatogonia Nuclei; M-Mitochondria). (**B**) The testis of GSP alone treated rat, show normal Sertoli cells, spermatogonia nuclei (N), basement membranes (Bm), and the inner most layer of sperm cells. (**C**) Image of Cd alone treated rat displaying irregular membrane of the spermatogonia nucleus with vacuolated cytoplasm where endoplasmic reticulum found to dilate and mitochondria is seeming to be swollen and vacuolated, (N-Spermatogonia nucleus). (**D**) Pre-administration of GSP to Cd-treated rats’ shows a significant recovery which is supported by the normal spermatid nucleus, endoplasmic reticulum, and mitochondria, (N-Spermatid Nuclei) (bars 2 µm) (×12000).

Electron microscopic examination is showed in Figure 10, in control rats (Figure 10A) and GSP treated rats (Figure 10B). Electron micrographs showed the normal structure of seminiferous tubules. Sertoli cells were identified by their large indented nuclei, numerous mitochondria, rough endoplasmic reticulum (RER), spermatocytes were seen. In Cd-treated rats extensive testicular tissue damage, inflammatory changes appeared in the form of thickened and irregular endothelial and basement membranes with vacuolar degeneration (Figure 10C). Sertoli cells and spermatocytes showed blebbing of the membranes with cytoplasmic vacuolation, together with chromatin condensation and margination in primary spermatocytes. GSP pre-treatment (Figure 10D) restored the normal electron microscopic appearance of testicular tissue similar to that observed in the control group (Figure 10A). The endothelial and basement membrane were regular and of normal thickness. Normal embedding of spermatids in Sertoli cells and normal acrosomal granules were observed without cytoplasmic vacuolation or abnormal chromatin distribution.

## Discussion

The present study reports the potential protective effects of GSP against Cd-induced toxicity by studying the changes in seminiferous tubules and testicular interstitium. The current study has shown reduced male fertility, such as decreased sperm count and poor semen quality, in men exposed to Cd and/or other environmental toxicants [34]. Cd that tends to accumulate in the testes has attracted substantial interest, Cd induced testicular toxicity involves the inhibition of oxidative stress, leads to an increase in germ cell apoptosis and BTB impairment to subsequent germ cell loss, edema, and hemorrhage in testis. Cd shows gonadotoxic and spermiotoxic potentials and exerts adverse effects on reproductive structures and function at the testicular level by altering the post-testicular events such as sperm progress motility and viability which culminate hypogonadism and infertility [35]. The symptoms of Cd toxicity in rats include: (i) body weight retardation in rats, which could be due to tissue damage and reduction in their functions. The mass of the undifferentiated spermatogenic cells also reflects the weight of the testicular tissues. (ii) Reduction in epididymal sperm concentration, (iii) suppression of sperm progress motility, (iv) increase in both percent total sperm abnormalities and live/dead count.

In our study, we examined the effect of Cd on the testicular histology of the rats. Our results clearly showed that Cd severely damaged the seminiferous tubules and interstitium, including the congestion of vessels and hemorrhage, the formation of the vacuoles in epithelial cells, the desquamation of epithelial cells in the lumen, the disorder of seminiferous tubule germinal epithelium, multinucleated giant cells, and necrosis of some seminiferous tubules. The formation of multinucleated giant cells in Cd-intoxicated rats indicates the continuous degeneration of the spermatogenic epithelium and appears to represent a non-specific reaction to injury. The giant cells are proposed to be a result of the inability of primary spermatocytes to undergo meiotic divisions to generate haploid sperm cells, which undergo additional DNA replication giving rise to multinucleated giant cells [35]. Ultrastructural findings also reveal severe necrosis and degeneration of seminiferous tubules with complete loss of spermatogenic cell layers and absence of centrally located spermatozoa. The irregular membrane of the spermatogonia nucleus with vacuolated cytoplasm appeared where endoplasmic reticulum is found to dilate and mitochondria seemed to swell and vacuolated. Additionally, some of the seminiferous tubules showed central debris with damaged Sertoli cells. Our findings are consistent with other studies and further support their observations [36,37].

The testis weight loss due to the effect of Cd depends on the reduced tubule size, spermatogenic arrest, and inhibition of steroid biosynthesis of Leydig cells. The significant decrease in sperm motility and count is associated with the effect of Cd on germ cells and elongated spermatids [38]. Mature sperms are enriched with polyunsaturated fatty acids in which the oxidative stress is prominent, Cd increases the sperm membrane lipid peroxidation to impede the sperm progress motility and increase percent total sperm abnormalities as well as cause dramatic loss in the fertilizing potential of sperm. The present results showed that pre-administration of GSP had no toxic effect on rats, in case GSP significantly mitigated the effects of Cd on the sperm morphology, concentration, and sperm motility parameters, reduces the testicular damage with a significant restoration of body weight, testicular and epididymal weight. The rich sources of monomers such as catechin, epicatechin, dimeric, trimeric, and tetrameric proanthocyanidins molecules possess a structure that confers on them an antioxidant property, which has been demonstrated to exert a novel spectrum of biological, pharmacological, therapeutic, and chemoprotective effects against oxygen free radicals and oxidative stress [39].

Cd testicular toxicity is associated with apoptosis induction, although a few studies have shown the induction of apoptotic cell death following exposure to Cd alone via different cell death pathways [40]. Immunohistochemistry analysis showed the intensity of caspase-3 in the testes was significantly elevated by Cd toxication, GSP pretreatment on the contrary side significantly inhibited the Cd-induced expression of caspase-3, an executioner of cell apoptosis, in testicular tissue. The eNOS, iNOS, and eNOS are known to be associated with infertility, spermatogenesis, and sperm maturation of testis. The increased expression of eNOS in ischemia and reperfusion injury of the testis has been reported previously [36]. Kolluru et al. [41] reported that micromolar concentrations of Cd led to endothelial dysfunction by blocking eNOS activity. Similarly, we found a significant elevation in the expression of eNOS activity in testis of rats after Cd exposure. This could be due to interference of Cd with the calcium-associated NOS activity, causing alterations in the subcellular trafficking of eNOS. HSP70 plays a crucial role in cellular division and development during spermatogenesis process and is also involved in regulation of synthesis of steroid hormones in Leydig cells [34]. Previous studies on animal models showed that the absence of HSP70 in the germinal epithelium of mice resulted in triggering of apoptosis through P53-dependent mechanism [42]. In our study, we found a significant increase in HSP-70 expression in the testis of rats. Cd toxicity caused a significant increase in denatured or misfolded protein, thereby increasing the magnitude of Hsp70 response. On pretreatment with GSP, immunoreactivity of eNOS, HSP70, and caspase 3 in Cd-induced testes were significantly decreased. Thus, GSP can be considered as useful pharmacological agents to protect against Cd-induced testicular injury.

Oxidative stress plays a crucial role in the etiology of defective sperm function by involving the induction of peroxidative damage to the plasma membrane. Lipid peroxidation of membranes is regulated by the availability of Cd-induced free radicals and excited state molecules, to initiate propagation, the antioxidant defense status of the environment, and the physical status of the membrane lipids. The elevated level of lipid peroxidation products such as TBARS, LOOH, and PC in the testicular tissue of Cd-intoxicated rats in the present research noticeably discovered the oxidative stress and failure of the antioxidant system in it. Cd intoxication impedes an increase in hydroxyl radicals that can accelerate lipid peroxidation and this is attributed to the formation of peroxyl radicals that may react with the lipids possibly by hydrogen abstraction leading to male infertility [43]. GSP on the other side mediate through the modulation of cellular antioxidant levels in order to scavenge free radicals and lipid peroxides in the testes of rats. GSP acts as a powerful antioxidant, facilitates the detoxification process, thus minimizing Cd-dependent production of toxic free radical species along with drastically limiting the ongoing lipid peroxidation [44].

Glutathione is one of the essential compounds for regulation of a variety of cellular functions. It has a direct antioxidant function by reacting with superoxide radicals, peroxy radicals, and singlet oxygen followed by the formation of oxidized glutathione (GSSG) and other disulphides. Cd-induced oxidative stress is strongly suggested with the decreased levels of reduced GSH, TSH, vitamin C and vitamin E. The depletion of the testicular GSH in the present study is one of the factors responsible for enhanced oxidative damage to proteins and lipids. In Cd exposed tissues GSH decreases and forms a complex between Cd and GSH (Cd- GSH) via a reaction catalyzed by GST [45]. Thus the increased GST activity under Cd influence may be a defensive mechanism toward the free radical damage to tissues. Vitamin E takes a protective role in integrating the cellular membranes from oxidative damage while as vitamin C appears to be the prime contour of antioxidant defense. During Cd intoxication, the levels of vitamin C and vitamin E were decreased, which also contributed to the development of Cd-induced testicular damage. GSP on the other side has multiple phenolic hydroxyls which favor the interactions with biological membranes that can occur via the formation of hydrogen bonds. GSP has both hydrophobic and hydrophilic residues within the flavan-3-ol molecule, which interacts with the phospholipid head groups that results in changes in a number of membrane properties leading to alterations in the regulation of membrane-bound enzymes and receptors [46,12].

Oxidative stress is a common factor regarding male infertility, illustrating the importance of Cd as an inducer of oxidative stress [36]. The antioxidant enzymes such as SOD, CAT, GPx, GST, and G6PD constitute the secondary line of enzymatic antioxidant defense against ROS-induced oxidative stress. Cd decreases their concentration with significant reduction in testicular function and androgen secretion with the result induced oxidative stress by Cd either leads to the oxidative damage or activates signal transduction pathways to initiate defense responses. Turn down activity of these enzyme levels may be explained by the fact that excessive superoxide anions may inactivate SOD thus, resulting in inactivation of the H_2_O_2_ scavenging enzymes. Pretreatment of GSP significantly restored the antioxidant enzyme defense in as exposed rat testes via its strong antioxidant property. GSP might decrease the workload of SOD, CAT/GPx, and reduce the free radical mediated inactivation of enzymes and thereby sustain the activities of enzymatic antioxidant in testis.

The Cd-exposure effects decrease the total ATPase, Na^+^/K^+^-ATPase, Mg^2+^-ATPase, and Ca^2+^-ATPase activities. These enzymes are highly vulnerable to peroxidation by the action of lipid peroxidation and are involved in the translocation of sodium, potassium, calcium, and magnesium ions across the cell membranes. The decreased activities of ATPases in Cd-intoxicated rats could be the result from the formation of Cd–ATPase complexes through –SH group of enzymes and increased oxidative stress. GSP stabilizes the membrane due to its property of inhibiting the lipid peroxidation in cell membranes in the testis which abolished the Cd-stimulated elevation of intracellular free Ca^2+^ concentration and restored the cadmium induced inhibition of Na^+^, K^+^, Mg^2+^, and Ca^2+^ ATPases.

Testis is considered as an immune privileged organ under which inflammatory cytokines are one of the most compelling etiological features of male infertility. Cytokines are known to regulate multiple physiological functions including germ cell development, Leydig cell steroidogenesis, and extracellular matrix (ECM) biosynthesis in the testis. TNF-α is produced by Sertoli and germ cells in the testis and is a crucial cytokine that regulates a wide range of cellular processes in the testis [47]. Cd showed the increased concentration of TNF-α, IL-1β, IL-6, IL-10, iNOS, NO, and IFN-γ in the testis of rat due to the increased oxidative stress, which can stimulate germ cell apoptosis and disrupt Sertoli tight junction integrity and germ cell attachment. A large proportion of NO in the testes is produced by iNOS derived from testicular macrophage. Our results suggested that GSP could effectively suppress the activities of inflammatory cytokines through its strong antioxidant ability. GSP inactivate the synthesis of IL-10 to counteract the action of this anti-inflammatory cytokine. GSP could have a direct negative transcriptional effect on the iNOS gene resulting in lower levels of NO, which represents one of the key elements involved in the testis pathology.

Transaminases are the most sensitive biomarkers directly implicated in the extent of cellular damage and toxicity because they are cytoplasmic in location and are released into the circulation after cellular damage [48]. Testicular biomarker enzymes (AST, ALT, ACP, ALP, and LDH) play an important role in testicular metabolism, differentiation, and proliferation of germinal epithelium and transporting materials from Sertoli cells to various germinal cells. In Cd-treated rats the increased activity of testicular phosphatase and ACP reflects the testicular degeneration, which can be an indication of lytic activity. The lysosomal breakdown of testicular cells or to increase phagocytic activity of the Sertoli cells is the main reason for the increased activity of ACP in Cd-treated rats. The alterations in enzyme activity may lead to the destruction of the seminiferous epithelium and loss of germinal epithelium, resulting in the reduction in the number of spermatids associated with the decrease in sperm production in the testis. GSP regulates the activities of those enzymes by protecting free radicals responsible for oxidative stress and the production of ROS as supported by restoration of non-enzymatic and enzymatic antioxidants in the testes. GSP effectively stabilizes the spermatogenic development and spermatogenesis via its antilipidperoxidation activity and it is supported by the reduced levels of ALT, AST, ALP, ACP, and LDH in testes of GSP pretreated rats.

Testicular androgenesis is controlled by two rate limiting steroidogenic enzymes (Δ^5^ 3β-HSD and Δ17β-HSD), which are essential and limiting factors in the testicular testosterone synthesis plus is responsible for the transport of cholesterol into mitochondria [49]. In our study, these enzymes were decreased, which might be due to decrease in cytochrome (P450scc) and a gradual decrease in *3β-HSD* mRNA levels in the testis of Cd-treated rats [50]. Due to the inhibition of these testicular androgenic enzyme activities, the testosterone is decreased in Cd-treated rats; our results are inconsistent with [50]. Cd toxicity has been related to the decreased Leydig cell viability, which leads to the decreased testosterone secretion which in turns stimulates the release of gonadotrophin releasing hormone from the hypothalamus. An increased Cd accumulation in testis and increased levels of FSH, LH and prolactin in rats, suggesting a possible effect of Cd on the testicular axis testosterone. Pretreatment of GSP in Cd-treated rats increased the level of LH, FSH, and steroidogenic enzyme concentration by directly up-regulating hormone synthesis in addition to protect the synthetic pathway from oxidative inhibition, showing its ability to protect the male fertility damaged by Cd as it can scavenge the free radical and ROS induced by Cd.

Cd induced ROS and oxidative stress have been associated with apoptosis in many cell types including spermatogenic cells. Cd intoxication has been shown to increase the expression of pro-apoptotic protein Bax and caspase 3, while reducing the expression of anti-apoptotic protein Bcl-2. Cd-induced apoptosis showed the down-regulated expression of p-Akt and PI3K, which was found higher in GSP. p-Akt causes the phosphorylation of BAD, but when pAkt is suppressed, BAD remains dephosphorylated. Dephosphorylated BAD forms a heterodimer with Bcl-2 and displaces Bax. Increased Bax by Cd promotes apoptosis by heterodimerizing with Bcl-2. The alteration of the Bax to Bcl-2 ratio appears to determine whether some cells live or die. GSP pretreatment effectively suppressed the activation of caspase 3 and Bax, activates anti-apoptotic Bcl-2 via modulation of both mitochondrial and cytosolic antioxidant enzyme systems with an increase in GSH and protects the testis from Cd-induced ROS attack.

During Cd-induced oxidative stress, cysteine residues in Keap1 become oxidized, and this modification changes the conformation of the whole complex (Nrf2–Keap1). Nrf-2 escapes the Keap1-dependent cytoplasmic sequestration and degradation, leading to stabilization of Nrf2, increased nuclear accumulation of Nrf2, and activation of Nrf2-dependent cytoprotective genes [51]. Pre-administration of GSP significantly inhibits the Keap1 protein and modulates the expression of Nrf-2, HO-1, γ-GGL, and NQO1 through the expression of the phase II detoxifying and antioxidant enzymes such as NADPH and HO-1. In the present study, we found pretreatment of GSP up-regulates the level of PI3K in Cd exposed cells. Hence, it could also possibly up-regulate the expression of Nrf-2. GSP with the activation of PI3K/Akt pathway regulates the level of Nrf2-dependent inducible expression of HO-1 and correlates with a noticeable increase in Nrf-2 expression in testis of rats.

DNA is very sensitive to the effects of oxidative stress. Especially, oxygen-derived free radicals like OH• cause formation of a number of modified bases and sugars in DNA. In the present study, Cd inhibits nucleotide excision repair involved in the removal of a broad spectrum of DNA lesions induced as the repair of oxidative DNA damage. Cd performs the inhibition of repair effect by replacing with zinc, which takes part in the structure of the repairing enzymes. It is expected that this replacement might have a role in the *in vivo* carcinogenecity of Cd [52]. GSP protected the DNA oxidative damage and DNA fragmentation induced by Cd. One possible mechanism is that proanthocyanidins present in the GSP would allow the interception of free radicals generated by Cd before they reach DNA and presume contribution to DNA repair.

## Conclusion

To summarize, the results of the present study suggested that GSP afford therapeutic and prophylactic efficacy against Cd induced oxidative testicular toxicity through its strong antioxidant property. GSP ameliorated the Cd induced alterations in testis through the activation of Nrf2 by PI3K/Akt mediated pathway. Moreover, GSP suppresses the activation of apoptosis via modulation of cytosolic antioxidant enzyme system in the testis. The antioxidant, anti-inflammatory, and anti-apoptotic properties could be considered as the principal factors responsible for the testicular protection afforded by GSP. Combining, we state that further research may establish this bioactive proanthocyanidin, GSP, as a possible candidate for the treatment of Cd-induced oxidative stress associated testes complications in near future.

## Abbreviations

ACP: acid phosphatase
Akt: Protein kinase B
ALP: alkaline phosphatase
ALT: alanine aminotransferase
AST: aspartate aminotransferase
Bcl2: B-cell lymphoma2
BTB: blood–testes barrier
BW: body weight
Cas-3: Caspase-3
CAT: catalase
CCD: Charge coupled device
CD: Conjugated dienes
Cyt c: Cytochrome complex
DCF-DA: 2′,7′-dichlorofluorescin diacetate
DPBS: Dulbecco’s PBS
eNOS: endothelial nitric oxide synthase
FSH: follicle stimulating hormone
γGCL: gamma glutamate cysteine ligase
γGCS: gamma glutamyl cysteine synthetase
GPx: glutathione peroxidase
GR: glutathione reductase
GSH: glutathione reduced
GSP: grape seed proanthocyanidins
GST: glutathione S-transferase
G6PD: glucose-6-phosphate dehydrogenase
HO1: Heme oxygenase1
HSP70: heat shocking protein70
IFN-γ: interferon-γ
IL: interleukin
iNOS: inducible nitric oxide synthase
I.P: Intra peritoneal
Keap-1: Kelch like ECH associated protein1
LDH: lactate dehydrogenase
LD50: Lethal dose50
LH: leutinizing hormone
LOOH: Lipid hydroperoxides
NQO1: NAD(P)H quinone dehydrogenase1
Nrf-2: Nuclear factor erythroid 2- related factor 2
PC: Protein carbonyls
PI3K: Phosphoinositide 3 kinase
ROS: reactive oxygen species
SOD: superoxide dismutase
TBARS: Thio barbituric acid reactive substances
TNF-α: tumor necrosis factor-α
TSH: total sulphydryl group
UGC: University grants commission
3β-HSD: 3β-hydroxysteroid dehydrogenase
17β-HSD: 17β-hydroxysteroid dehydrogenase
