# Expression of Concern: The molecular and biochemical insight view of grape seed proanthocyanidins in ameliorating Cadmium-induced Testes-toxicity in rat model: Implication of PI3K/Akt/Nrf-2signaling

**DOI:** 10.1042/BSR-20180515_EOC

**Published:** 2020-08-04

**Keywords:** cadmium, oxidative stress, antioxidant, grape seed proanthocyanidins, Nrf2/HO-1, testes

The Editorial Office has been made aware of potential issues surrounding the scientific validity of this paper, hence has issued an expression of concern to notify readers whilst the Editorial Office investigates.

